# Combined DFT-D3 Computational and Experimental Studies on g-C_3_N_4_: New Insight into Structure, Optical, and Vibrational Properties

**DOI:** 10.3390/ma16103644

**Published:** 2023-05-10

**Authors:** Paolo Negro, Federico Cesano, Silvia Casassa, Domenica Scarano

**Affiliations:** Department of Chemistry and NIS (Nanostructured Interfaces and Surfaces) Interdepartmental Centre, University of Torino & INSTM-UdR Torino, Via P. Giuria 7, 10125 Torino, Italy; federico.cesano@unito.it (F.C.); silvia.casassa@unito.it (S.C.)

**Keywords:** graphitic carbon nitrides, melon polymorph structure, DFT-D3 computation, structure and morphological properties, optical properties, vibrational properties

## Abstract

Graphitic carbon nitride (g-C_3_N_4_) has emerged as one of the most promising solar-light-activated polymeric metal-free semiconductor photocatalysts due to its thermal physicochemical stability but also its characteristics of environmentally friendly and sustainable material. Despite the challenging properties of g-C_3_N_4_, its photocatalytic performance is still limited by the low surface area, together with the fast charge recombination phenomena. Hence, many efforts have been focused on overcoming these drawbacks by controlling and improving the synthesis methods. With regard to this, many structures including strands of linearly condensed melamine monomers, which are interconnected by hydrogen bonds, or highly condensed systems, have been proposed. Nevertheless, complete and consistent knowledge of the pristine material has not yet been achieved. Thus, to shed light on the nature of polymerised carbon nitride structures, which are obtained from the well-known direct heating of melamine under mild conditions, we combined the results obtained from XRD analysis, SEM and AFM microscopies, and UV-visible and FTIR spectroscopies with the data from the Density Functional Theory method (DFT). An indirect band gap and the vibrational peaks have been calculated without uncertainty, thus highlighting a mixture of highly condensed g-C_3_N_4_ domains embedded in a less condensed “melon-like” framework.

## 1. Introduction

The prospect of developing inexpensive, energy-efficient, green, and sustainable industrial processes has attracted extensive interest in the scientific and industrial communities. For this reason, in the past few years, among the carbon-based materials, g-C_3_N_4_, a polymeric metal-free semiconductor photocatalyst with 2D graphene-like structure, has received massive attention [1,2,3,4,5,6,7]. Due to its exceptional properties, including easy preparation methods from economical raw materials (i.e., earth-abundant carbon- and nitrogen-based materials), high thermal and chemical stabilities, non-toxicity, tunable band gap, and visible light absorption, it turns out that widespread applications, such as the removal of toxic metal ions, degradation of dye pollutants, selective organic transformation to fine chemicals, and photocatalysis for hydrolytic hydrogen production, have been developed [8,9,10,11,12]. Moreover, it can be used in additional fields such as metal-free organic syntheses [13,14,15,16,17,18,19,20], solar-light-driven photo-redox catalysis, photocatalytic water splitting, photoelectric conversion, and fuel cells [9,12].

It is well known that g-C_3_N_4_ is a n-type semiconductor, the framework of which is formed of π-conjugated N-bridged aromatic poly tri-s-triazine units (heptazine rings) forming layers with strong C-N covalent bonds. Namely, it was found that the 2D lattice consists of a periodic arrangement of nitrogen-linked heptazine units, in which each nitrogen connects two or three heptazine rings, thus giving rise to a variety of structural motifs with different levels of condensation, ranging from partially to fully polymerised structures [21,22]. The π-conjugated sp^2^ hybridised structure is also responsible for the visible light absorption at about 460 nm, which explains the typical pale yellow colour [23].

Regarding the thermal properties, g-C_3_N_4_ is highly stable up to 600 °C, with C-N fragmentation reactions occurring at higher temperature, while concerning the chemical stability, the van der Waals interactions among layers render it non-reactive to many conventional solvents [13].

However, some drawbacks must be mentioned, including the low specific surface area, the chemical inertness itself, the poor charge carrier mobility, and the high exciton recombination rates. Nevertheless, since g-C_3_N_4_ has been considered a promising/challenging material in many fields [24,25,26], to overcome the aforementioned drawbacks, and also to improve its already existing properties, g-C_3_N_4_ has been combined often with metals or other semiconductors to form heterojunctions or composites with innovative architectures, aiming at enhancing the charge carrier rates, the surface properties, the surface area, etc. [27]. Concerning the innovative architectures, low dimensional carbon nitride nanostructures, i.e., 1D nanowires, nanotubes, or nanorods, as well as 2D nanosheets, show improved charge transfer ability compared to the bulk structures. Furthermore, porous 3D nanospheres provide an increased specific surface area, thus also improving adsorption ability and, ultimately, the photocatalytic performance [21,28]. In this respect, detailed investigations on the surface properties of carbon nitride structures with different polymerisation degrees have been carried out to understand the role of terminal amino groups and (C=N–C) neighbouring triazine nitrogen units in catalytic reactions as potential active sites to form heterostructures [29].

It is known that, due to the commonly used synthesis methods (thermal polycondensation in air), high crystalline g-C_3_N_4_ structures are difficult to obtain [3]. This means that the peculiar properties of g-C_3_N_4_ itself depend strongly on the experimental conditions, i.e., on the different precursors that are used during the synthesis, on the reaction rates, and/or temperatures, thus affecting the local structure, the stacking order, and the formation of defective situations, which play a significant role in modifying the band structures [5,23].

On this matter, several computation models have been proposed and compared with the experimental data by changing the stacking order and the inter-layer and inner-layer distances between heptazine chains, together with, in some cases, ring-opening reactions, which give rise to nitrogen defects, thus increasing the complexity of the models [6,30,31,32,33]. Despite the many efforts focused either on experimental (i.e., neutron and X-ray diffraction patterns) and on computational approaches [3], which predict amazing properties and applications, the complex structure of the polymeric carbon nitride systems is still poorly understood, being, therefore, also a matter of a controversial debate [14,34,35].

Along this theme, our aim is to give some insights into the more suitable carbon nitride model with a stable configuration, which is able to describe properly the experimental results obtained from X-ray diffraction patterns, UV-visible, and FTIR spectra. Therefore, two structures with two different degrees of condensation of g-C_3_N_4_ systems have been investigated and characterised at the Density Functional Theory level (DFT) by means of the periodic simulation code CRYSTAL [36]. Indeed, interestingly, a realistic model that is able to explain the experimentally measured properties is based on the coexistence of different structural motifs. As previously stated, it is challenging for the adopted approach to shed light on the electronic structure of both partially (melon-like) and fully (g-C_3_N_4_) polymerised systems as such, with the aim of developing more complex heterostructures such as homo–heterojunctions, which could enable a more effective light absorption with the consequent use in visible-light-responsive photocatalysis.

With regard to this, it is well known that DFT calculations can also provide a description of the valence and conduction bands in terms of atomic orbitals, which potentially play a relevant role as oxidation and reduction sites in photocatalysis [2].

## 2. Computational Set Up

Throughout this work, we apply DFT as implemented in CRYSTAL17 [36], a periodic quantum-mechanical code based on the description of the crystalline wave function as a linear combination of localised Gaussian-type atomic orbitals.

A 6-21G basis set plus polarisation functions (3-1G for hydrogen atoms), which is used extensively for solid-state system calculation, was adopted for C and N atoms and will be referred to in the following sections as 621pol [37]. Functionals with different percentage of Hartree–Fock (HF) exchange have been explored, including pure Perdew–Burke–Ernzerhof functional (PBE) [38], PBE0 (25% of exact change) [39,40], and HSE06 [41] (Table S1). The calculations were finally performed at the PBE/621pol level. Moreover, since the van der Waals interactions play a fundamental role in establishing these materials, the PBE was enriched with the D3 a-posteriori empirical correction proposed by Grimme [42] to account for the London dispersion forces (PBE-D3).

The DFT exchange-correlation contribution was evaluated via numerical integration over the unit cell volume by using a pruned grid with 99 radial and 1454 angular points. Integration over the reciprocal space was carried out by using Pack–Monkhorst meshes of 4 × 4 × 4. The Coulomb and exchange series, which were summed in direct space, were truncated by using overlap criteria thresholds of *[7, 7, 7, 12, 24]*. Convergence for the self-consistent field algorithm was achieved up to a threshold of 10^−9^ Hartree on the total energy, per unit cell [43].

Geometry optimisation was performed by using analytical gradients with respect to atomic co-ordinates and unit cell parameters, within a quasi-Newtonian scheme combined with Broyden–Fletcher–Goldfarb–Shanno Hessian updating [44,45]. The default convergence criteria were adopted for both gradient components and nuclear displacements.

A full set of vibrational frequencies in k = Γ was obtained within the harmonic approximation by diagonalising the mass-weighted Hessian matrix. This matrix was built by numerically differencing the analytical gradient with respect to atomic cartesian co-ordinates [46,47].

The topological analysis of electron density, which allows an accurate mapping of bonds and atoms, was carried out by means of the TOPOND [48] program, which was recently incorporated in the CRYSTAL [48,49,50] code. The topological properties of the critical points of the bonds were analysed according to Bader’s theory of atoms in molecules and crystals (QTAMAC) [51] to acquire information on the number and type of interactions.

## 3. Materials and Methods

**Scheme 1:**
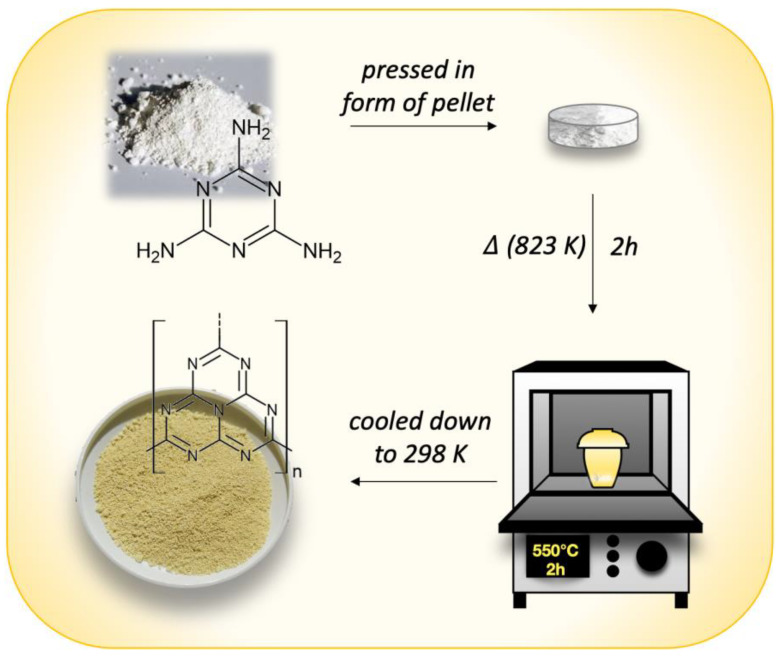
Main steps of g-C_3_N_4_ synthesis.

Graphitic carbon nitride (g-C_3_N_4_) was prepared by heating 2 g of melamine powder (Sigma-Aldrich, Burlington, MO, USA, Melamine 99% M2659) inside a muffle (Nabertherm B400). In more detail, to avoid the melamine leak during the precursor sublimation, the powder was pressed into the form of a pellet and introduced in a ceramic crucible, which was closed with a ceramic lid (even if not-hermetically). The sample was heated up to a temperature of 823 K with a heating rate of 1 K per minute and kept for 2 h at the same temperature. Then the sample was slowly cooled down to room temperature and weighed (resulting in about 48% of the initial mass) (see Scheme 1).

From the photograph in Scheme 1, it appears that the obtained material on the white sample holder shows a yellowish colour, which makes it possible to predict the energy value of the absorption edge in the UV-Vis diffuse reflectance spectra (vide infra).

The X-ray diffraction patterns were obtained by using a PANalytical PW3050/60 X’Pert PRO MPD X-ray diffractometer with a Cu radiation (K𝛼 = 1.54060 Å) and a Ni filter in Bragg–Brentano configuration, which was equipped with a X’Celerator detector. The diffractograms were acquired in the 5° ≤ 2θ ≤ 80° interval with an acquisition step of 0.01°.

N_2_ adsorption–desorption experiments were carried out at 77 K by a Micromeritics ASAP 2020 instrument (Micromeritics, Norcross, GA, USA) to determine the Brunauer–Emmett–Teller (BET) surface area. The surface area of the samples was determined after outgassing at 423 K, overnight.

Scanning Electron Microscopy (SEM) analyses were carried out by using a ZEISS EVO 50 XVP microscope with a LaB_6_ source. Before performing the analysis, the insulating samples were covered with a gold layer of about 15 nm in thickness to avoid any charging effect (Bal-tec SCD050 sputter coater).

Atomic Force Microscopy measurements were carried out in the Intermittent-Contact (IC) mode by using a modified Nanosurf Easyscan2 AFM instrument, which was equipped with a 10 µm scan-head, inside a shielded and acoustically insulated enclosure on a high-performance anti-vibration platform.

The optical properties were investigated in the 190–2500 nm wavelength range by means of a Cary 5000 UV-vis-NIR spectrophotometer, which was equipped with a diffuse reflectance sphere in the diffuse reflectance (DR) mode. The sample was diluted in PTFE powder (2% by weight).

FTIR spectra were acquired in transmission mode by means of a Bruker VECTOR22 spectrometer that was equipped with a cryogenic MCT detector with 2 cm^−1^ resolution. Due to the high absorbance of the sample in the IR spectrum, the material was diluted in KBr (2% by weight). Each sample was pressed in the form of self-supporting pellets and then analysed under vacuum at 298 K.

## 4. Results and Discussion

### 4.1. Crystal Structure by XRD Analysis and Modelling

#### 4.1.1. Experimental XRD Analysis

**Figure 1:**
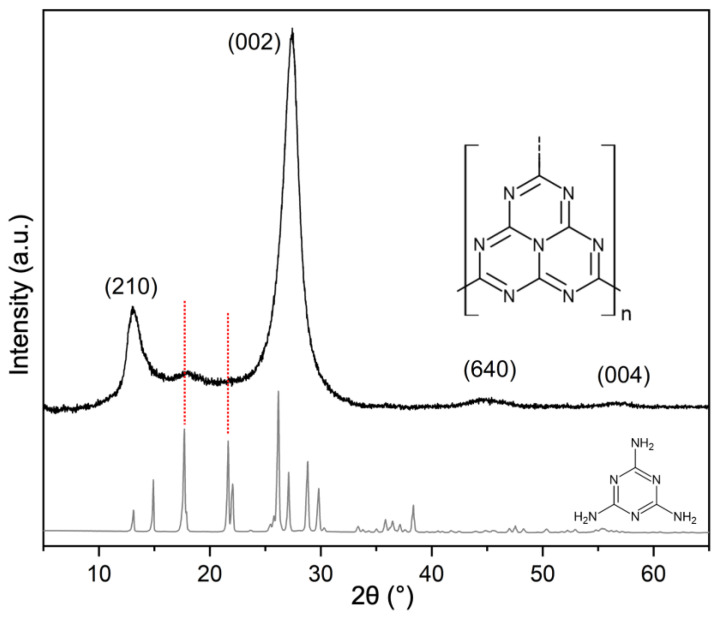
XRD patterns of the synthetised sample (black line) and of melamine (grey line). Red dotted lines highlight the presence of weak melamine features in the diffraction pattern of our sample.

From the X-ray diffraction pattern of our specimen (Figure 1, black line), two main peaks at approximately 13.04° and 27.6° are both assigned to layered g-C_3_N_4_ systems, in agreement with the literature data [7,52,53,54,55,56]. In particular, the feature at 27.6° (002), which is typical of the layered structure, is due to the inter-layer π–π stacking with a distance d(002) = 3.218 Å.

The feature at 2θ = 13.04° represents the in-plane packing of heptazine units and could be assigned either to the (210) diffraction planes of an orthorhombic unit cell [7] or to the (100) diffraction planes of a hexagonal unit cell [52,53,54,55].

For the sake of comparison, the XRD pattern of pure melamine powder has been shown (Figure 1, grey line). Due to the presence of broad and weak reflections at approximately 2θ = 17.7° and 2θ = 21.9° on the pattern of our specimen (red dotted lines), which are typical of melamine, it can be reasonably hypothesised that a network of hydrogen-bonded melamine/triazine units is present inside the heptazine framework.

From Scherrer’s equation D = kλ/βcosθ, where λ is the X-ray wavelength, β is the FWHM of the diffraction line corrected by the instrumental broadening, θ is the diffraction angle, and K is Scherrer’s constant or shape constant, which has been assumed to be 0.9, in agreement with Alizadeh et al. [57], the mean crystallite thickness of the particles has been calculated. In particular, from the (002) XRD peak (i.e., 2θ = 27.6°), the estimated dimension of the scattering domains is about 48 nm, in agreement with the BET surface area (about 9 m^2^/g).

#### 4.1.2. Modelling the Crystal Structure

**Figure 2:**
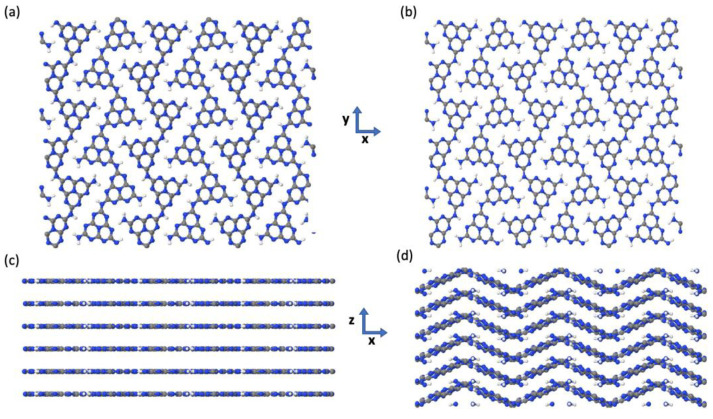
Melon structure before ((**a**,**c**) panels) [7] and after geometry optimisation ((**b**,**d**) panels). Top-view: (**a**,**b**) panels; side-view: (**c**,**d**) panels, obtained with Jmol program [58].

In this work, among the many structures proposed for carbon nitride polymerised structures, including g-C_3_N_4_, which was subjected to extensive and controversial discussions [1,2,3,4,5,6,7], we adopted the model proposed by Fina et al. based on their XRD and neutron diffraction analyses [7], which are reported for the sake of comparison in the a and c panels of Figure 2.

After a complete geometry optimisation at the PBE-D3/621pol level, the system produced results as in the b and d panels of Figure 2.

The peculiar intra-layer melon-like structure is retained, but the interplanar distance decreases, and a remarkable zig-zag layer deformation occurs. The data, which is reported in Table 1, confirm the aforementioned structural relaxation, which causes a contraction of the a, along the z direction, together with a slight increase in the b and c parameters with respect to the experimental values. As a result, a decrease in the total volume of the unit cell of about 5% is obtained.

The degree of distortion from the planar geometry can be estimated by comparing the bending angle defined as the angle among three in-plane nitrogen atoms univocally identified in Figure S1 of the Supporting Information. The magnitude of this angle changes from 175° in the experimental geometry to 151° in the optimised one.

It is worth noting that the zig-zag layer deformation is also evident at the PBE level, as shown in Figures S1 and S2 of the Supporting Information, and, therefore, cannot be an artifact due to an overestimation of the dispersive interactions between layers due to the D3 correction.

Thus, a notable rumpling of the layers characterises the equilibrium structure of the system calculated at zero Kelvin. The discrepancy between the calculated and experimental structures can be explained by invoking temperature effects. The refined structure, which was obtained at room temperature, could represent the average position of the vibrating lattice structure along the z-direction, above and below the plane around the equilibrium positions. If at T = 0 K, the interactions between the layers can freeze the structure in one of its lowest energy configurations, as the temperature increases, the kinetic degrees of freedom prevail, the structure vibrates, and the measurements provide the average position of the atoms.

To support the hypothesis of a stabilisation of the structure due to the formation of interactions between layers, we performed a comprehensive search of the charge density critical points and applied the full machinery of the topological analysis to characterise their nature.

In particular, bond critical points (BCP) were clearly revealed between hydrogen and nitrogen atoms belonging to neighbouring layers. Based on the values of the topological indicators (i.e., Laplacian of the charge density, virial density, ratio between total energy density and charge density), these interactions can be classified as hydrogen bonds, ref. [49] and could be responsible for the rippling of the planes. In addition, similar interactions, H-N, N-N, and C-N in type, were found between intra-layer atoms of neighbouring rows, and these can affect the lattice contraction. As expected, the structure expands due to the increase in temperature, and this explains the difference in volume between the experimental and the computed structures.

**Figure 3:**
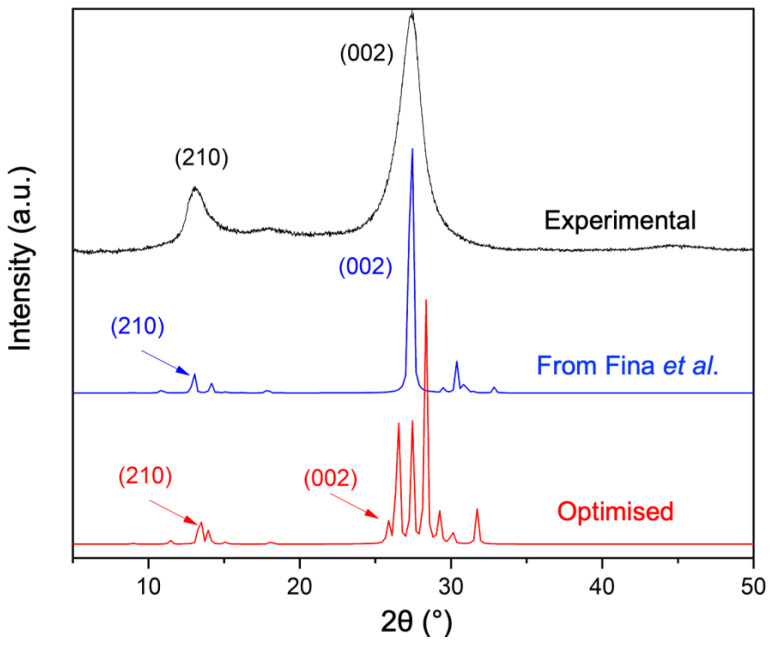
XRD diffraction patterns of the experimental structure (black line) compared with the XRD computed diffraction pattern before (Fina et al.) (blue line) and after PBE-D3/621pol optimisation (red line).

As a next step towards the atomic-scale insight into the synthesised sample, a close comparison among the XRD patterns was performed and reported in Figure 3. The XRD analysis computed on the geometry proposed by Fina et al., which is represented by the blue line in Figure 3, shows a good agreement with the pattern measured on our synthesised sample, black line, as both the characteristic peaks, due to the (210) and (002) diffractions, are clearly present. It is worth mentioning that the (002) signal is due to the inter-layer π–π stacking, while the (210) is related to in-plane packing of heptazine units. Thus, we can state that at least a partial condensation has been achieved and that our structure corresponds mainly to the melon polymorph with an orthorhombic space group P2_1_2_1_2.

Interestingly enough, the XRD pattern of the PBE-D3/621pol optimised structure (red line), shows, besides the features due to the (210) and (002) diffraction planes, additional intense components, within the 25° < 2θ < 35° range, due to different diffraction lattice planes caused by the rippling of the relaxed geometry.

Concerning the broadening of the experimental features (black line), we assume that it can be explained with an envelope of many XRD features that are reasonably related to different diffraction planes. This makes it possible to hypothesise the occurrence of differently oriented domains inside the synthesised sample. Therefore, for a complete interpretation of the XRD experimental results, it must be considered that a single model (partially polymerised melon) is not fully representative of the synthesised sample, but a total polymerised model can also be taken into account. According to this, a computational characterisation of a fully polymerised g-C_3_N_4_ structure becomes crucial to accurately reproduce the experimental result.

**Figure 4:**
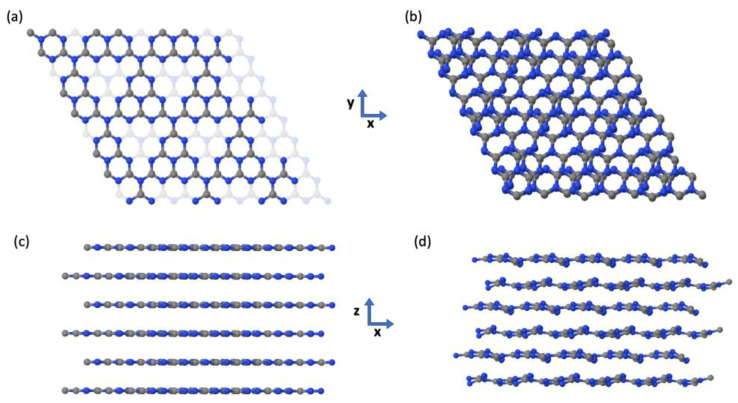
Fully condensed g-C_3_N_4_ structure before ((**a**,**c**) panels) [35] and after geometry optimisation ((**b**,**d**) panels). Top-view: (**a**,**b**) panels; side-view: c and d panels, which were obtained with the Jmol program [58].

The most plausible structure for representing the totally polymerised g-C_3_N_4_ has been proposed by Teter and Hamley [35] and belongs to the triclinic lattice, space group 1. In Figure 4, the experimental and PBE-D3/621pol optimised structures are shown for the sake of comparison, and in Table 2, the values of lattice parameters, volume of the reference cell, and the respective percentage variations are detailed. Although less evident with respect to the melon optimised structure, a layer rippling due to inter-/intra-layer interactions is observed on the relaxed structure (fully condensed g-C_3_N_4_ model, Figure 4b,d panels). Moreover, the bending angle between the N6, C21, and N3 atoms undergoes a variation of about 9% instead of 14%, as calculated for the partially condensed melon structure. Finally, a general shortening of the three lattice parameters (a, b, and c) that is accompanied by a decrease in volume of about 7% can be noticed.

**Figure 5:**
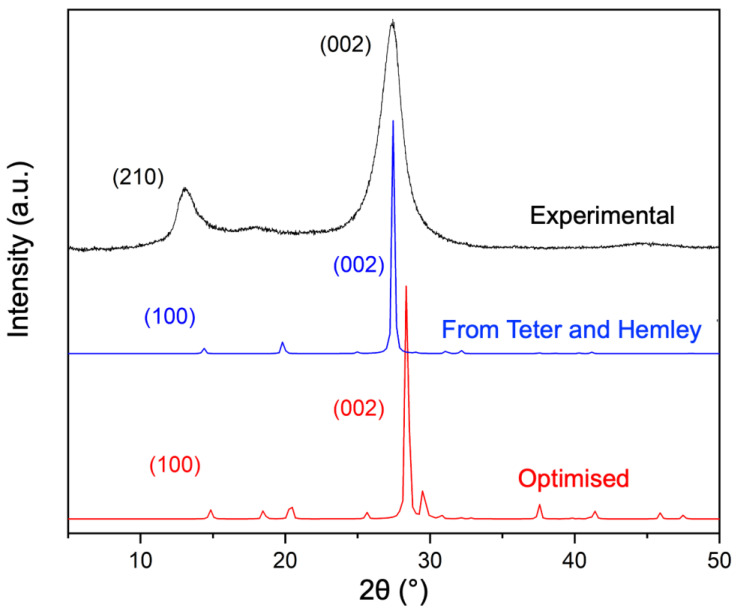
XRD diffraction patterns of the experimental structure (black line) compared with the XRD computed diffraction pattern before (Teter and Hemley) (blue line) and after PBE-D3/621pol optimisation (red line).

The diffraction patterns that were calculated for the Teter and Hemly geometry (blue line) and for the PBE-D3/621pol relaxed one (red line) are compared with the XRD pattern measured on our sample, and the agreement is again quite good. The peaks relating to the diffraction planes (002), (210), and (100) are present, although at slightly different 2 Theta values with respect to the experimental ones. In agreement with the decreased inter-layer distance of 4%, the position at higher angle values of the computed (002) diffraction angles (blue and red patterns in Figure 5) can be also reasonably explained.

A more effective comparison between the melon-like and totally polymerised structures discussed so far is shown in Figure S3 Supporting Information.

On the basis of the results discussed so far, we can definitely state that a realistic model, which is able to explain the experimental XRD patterns, implies the combination of different structural motifs including partially (melon-like) and fully polymerised g-C_3_N_4_ structures, as further shown in the following discussion, in agreement with Changbin Im et al. [22].

This result can find an explanation by considering the reaction equilibria along the slow pathway from melamine to fully polymerised C_3_N_4_. Indeed, the semi-closed reaction environment causes the formation of different volatile compounds including ammonia, which can stabilise several structures with a different polymerisation degree, including the melon-like structure, the fully polymerised C_3_N_4_, etc., with small differences in thermochemical stability, as confirmed by Changbin Im et al. [22]. (See reaction scheme in Figure S4 in Supporting Information). It is known that low temperatures and high p_NH3_ favour the formation of less condensed melon-string structures, whereas high temperatures and low p_NH3_ lead to more condensed g-C_3_N_4_ systems. Since these limited cases are not representative of the real situation inside the reaction environment, we expect that the obtained material consists of a mixture of less and more condensed structures [22].

### 4.2. Morphology by SEM and AFM Microscopies

**Figure 6:**
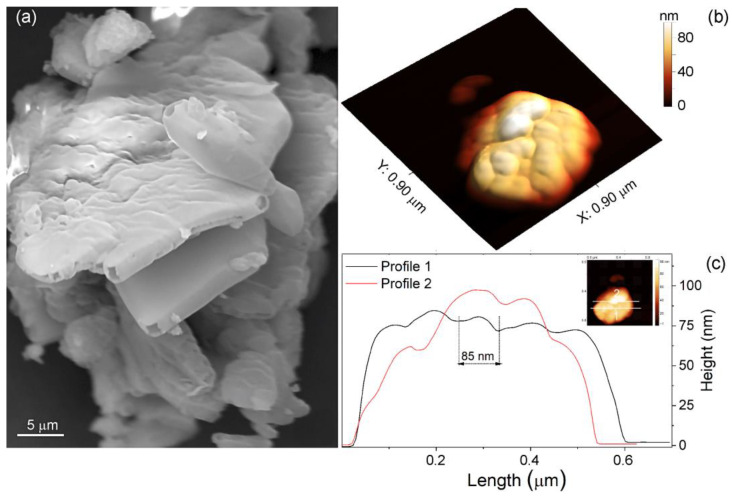
(**a**) SEM image, (**b**) 3D AFM image, and (**c**) AFM height profiles along two lines selected in the inset image, which is already shown in (**b**).

The morphology of the polymerised sample that is obtained from melamine, according to the previously described method, is SEM and AFM imaged in Figure 6.

From the SEM image (Figure 6a), a highly heterogeneous material, due to the several chemical processes occurring during the thermal treatment [59], including the decompositions and release of volatile compounds, has been obtained. In particular, complex aggregates of differently elongated rhombohedrons, besides lamellar and hollow structures, which are 5–15 µm in size, can be observed at the adopted resolution.

The high level of aggregation, together with the large size of the lamellar structures, in particular, can be explained with sintering effects due to slow synthesis steps, in agreement with the low specific surface area (see Paragraph 4.1.1).

In Figure 6b, the surface of a portion of g-C_3_N_4_ is 3D-AFM imaged on a flat freshly cleaved mica support that again confirms the formation of sintered structures, which are well adhering to each other and consist of large aggregates, as observed from the SEM image (Figure 6a).

Moreover, in Figure 6c, two height profiles along two directions on the inset image are shown. From the observed black and red profiles, the sizes of aggregated polymerised C-N nanoparticles can be estimated in the range of several tens of nanometres. Notice that the high degree of aggregation prevents us from evaluating the size of the single nanoparticles, or of the different domains as obtained from XRD analysis.

### 4.3. Optical and Electronic Properties Determined via UV-Vis Spectroscopy and DFT Calculations

The optical properties, as well as the electronic structure of the obtained specimen, have been investigated by means of UV-Vis spectroscopy and compared with DFT calculations.

**Figure 7:**
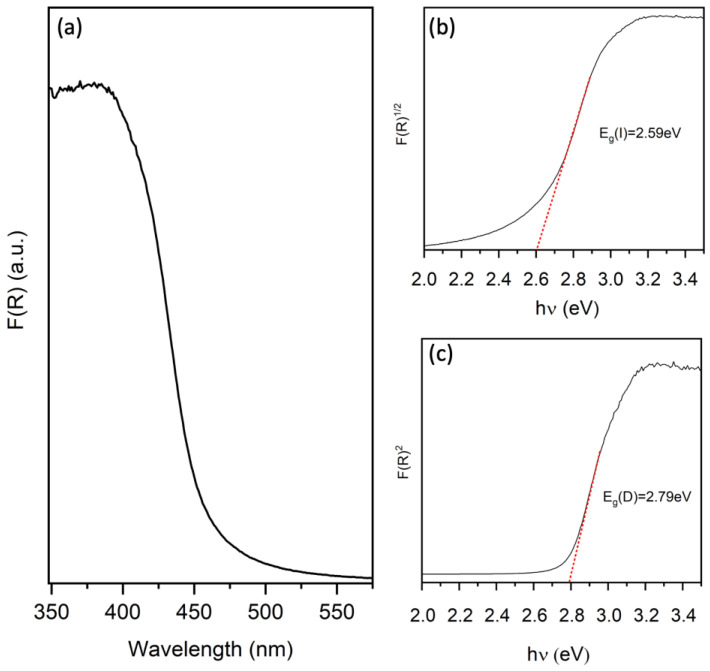
UV-vis spectra of g-C_3_N_4_ (**a**); the computed Tauc’s plots for indirect (**b**) and direct (**c**) band gap estimation.

In Figure 7a, the UV-visible spectrum is shown, together with the computed Tauc’s plots for indirect (Figure 7b, red dotted line) and direct (Figure 7c, red dotted line) band gap estimations.

From Figure 7a, the typical optical absorption edge of the polymerised carbon nitride system, due to the transition from HOMO N^3−^ 2p_x,z_ antibonding orbitals to the LUMO N^3−^ 2p_z_ and C^4+^ 2p_x,z_ lowest-energy orbitals (vide infra), has been observed, and the nitrogen and carbon p_z_ orbitals are available as oxidation and reduction sites for O_2_ and H_2_ evolution reactions, respectively [21].

Furthermore, a remarkable red shift towards longer wavelengths, with respect to melamine (Figure S5 in Supporting Information), can be detected as a result of the high polymerisation degree of our sample. This causes more delocalised π-electrons in the network, either inside the layers or along the stacking direction where the π-orbitals overlap [60]. In particular, from the evaluation of the optical band gap, the extrapolated onset, which is estimated via the Tauc’s plots, gives an indirect band gap value of 2.59 eV and a direct band gap value of 2.79 eV (Figure 7b,c, respectively). In the case of melamine, an indirect band gap of 4.71 eV and a direct band gap of 4.84 eV were obtained (Figure S3). Notice that the indirect band gap value of melamine is in agreement with that shown by Liu et al. (4.76 eV) [4]. The results are also consistent with those obtained via the theoretical calculations (vide infra). As a matter of fact, even if many studies have been focused on the investigations of the optical properties of g-C_3_N_4_, identifying the nature of band gap, whether it is direct or indirect, is not always straightforward. Therefore, for a deep understanding of the electronic properties of the material, an accurate description of the electronic band structures of both the partially and totally polymerised systems becomes crucial.

**Figure 8:**
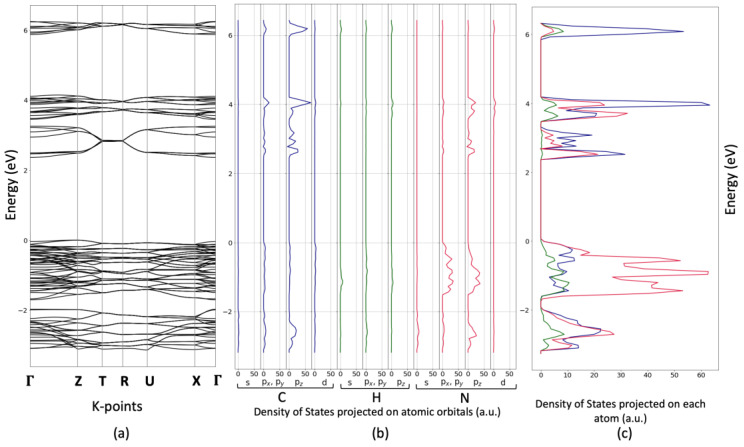
Band structure of partially polymerised (melon) system (**a**); projected density of states (PDOS) on the atomic orbitals (**b**); projected density of states (PDOS) on each atom (**c**). (Red lines for nitrogen, blue lines for carbon, green lines for hydrogen).

**Figure 9:**
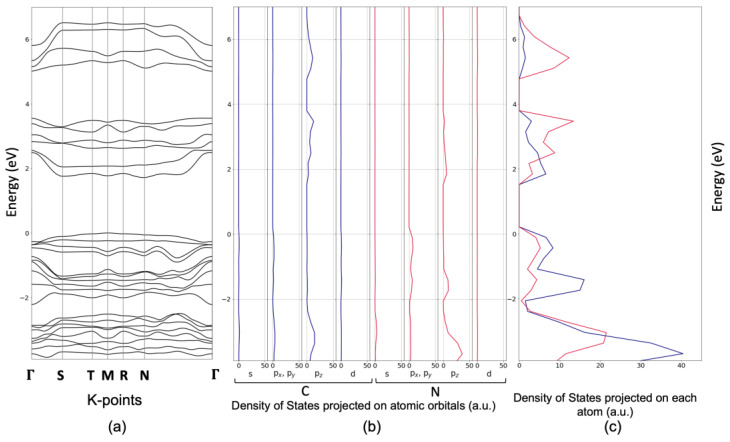
Band structure of fully polymerised g-C_3_N_4_ with the selected k-points: Γ (0,0,0), S (0,½,0), T (0,½,½), M (⅟4,½,½), R (½,½,½), and N (3/4,½,⅟4) (**a**); projected density of states (PDOS) on the atomic orbitals (**b**); projected density of states (PDOS) on each atom (**c**). (Red lines for nitrogen and blue lines for carbon atoms).

From the band structures shown in Figure 8a and Figure 9a, the semiconductor behaviour of melon and fully condensed g-C_3_N_4_ systems, respectively, can be highlighted. In particular, an indirect transition between the Z point at the top of valence band and the Γ point at the bottom of the virtual/conduction band of 2.36 eV and a direct transition from Γ (valence band) to Γ (conduction band) of 2.40 eV were computed for the partially condensed melon (Figure 8a). These results are in good agreement with the experimental estimation obtained from the Tauc’s plots (Figure 7b,c), and with a substantial amount of data reported in the literature [2,3,55,56,60,61,62,63], thus establishing that bulk structures show lower band gap than their few-layer or monolayer counterparts, which is expected to enhance the light-harvesting.

Concerning the totally condensed g-C_3_N_4_, an indirect transition from the N (3/4,½,⅟4) to the M (⅟4, ½, ½) point of 1.72 eV and a direct transition in the Γ point of 2.75 eV were computed. On the basis of these results, it becomes apparent that the optical properties of our material are better explained by the melon system, given that the computed energy gap is closer to the experimentally estimated one. However, the presence of some totally condensed g-C_3_N_4_ domains within the melon framework could not be excluded. Indeed, either the computed XRD or, mostly, the computed vibrational modes (vide infra), are in agreement with the experimental results, if a coexistence of both structures is taken into account.

Furthermore, the density of states that are projected on both the atomic orbitals and atoms (PDOS) of melon (Figure 8b,c panels, respectively) and of fully condensed g-C_3_N_4_ (Figure 9b,c panels, respectively) has been computed.

By comparing the panel a and b in Figure 8, it becomes apparent that the main contribution to the valence band of melon is mainly due to the p_z_ orbitals of nitrogen atoms, while the conduction band consists predominantly of carbon p_z_ orbitals, in agreement with Wang et al. [14]. This is further evident from the analysis of the projections on each atomic orbital in Figure 8c, in which the contribution of nitrogen (red lines) and carbon (blue lines) atoms to the valence and conduction band, respectively, are clearly remarkable. As expected, the hydrogen atoms (green line) have a negligible role in the PDOSs projection, even if their contribution in hydrogen bonding interactions between layers and rows is confirmed via the topological analysis of the electron density and supported by FTIR spectra (vide infra).

Concerning the valence and conduction band character, similar considerations can be extended to the total condensed system, except for the absence of hydrogen atoms together with functional end groups such as -NH (Figure 9b,c panel). Namely, the majority contribution to the valence band is given by nitrogen p_xy_ and p_z_ orbitals, while the conduction band comes mainly from carbon p_z_ orbitals.

### 4.4. Surface Vibrational Properties Determined via FTIR

**Figure 10:**
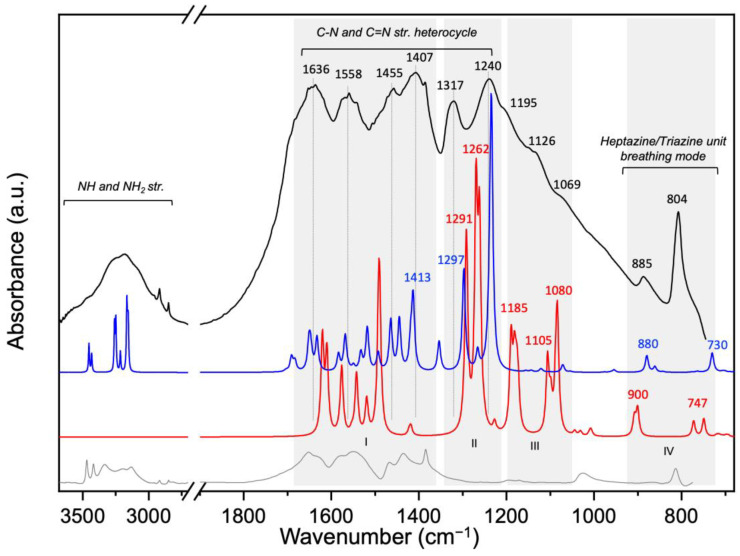
FTIR experimental spectra of our sample (black line) and melamine precursor (grey line); computed IR spectra of partially condensed melon (blue line) and fully condensed g-C_3_N_4_ (red line). The main regions of the spectra are highlighted by grey rectangles and labelled with Roman numbers from I to IV.

FTIR experimental (black line) and computed spectra of partially condensed melon (blue line) and fully condensed g-C_3_N_4_ (red line), together with the experimental spectrum of pristine melamine (grey line), as reference, have been shown in Figure 10.

The wide absorption band in the 3000–3500 cm^−1^ range of the experimental spectrum can be assigned to the stretching modes of NH/NH_2_ terminal groups of a melon-like structure [4,6,54,55,56,61,64,65,66,67], while the broadening is explained with H-bonding interactions [53,55]. This assignment is confirmed by the presence of the same features on the pristine melamine spectrum (grey line in Figure 10) [65]. From a close examination of the computed spectrum of melon, the main components inside the experimental convolution band can be identified as a combination of symmetric and asymmetric stretching modes of N-H and NH_2_ functional groups. Notice that due to the absence of NH/NH_2_ terminal groups in the fully condensed g-C_3_N_4,_ no spectral features are observed in the 3000–3500 cm^−1^ range, as expected.

Moving to the 1200–1750 cm^−1^ region (I and II in Figure 10), the typical vibrational modes of C-N heterocycles are found, as also confirmed via the theoretical computation [62]. In particular, the absorptions at 1636 cm^−1^, 1558 cm^−1^ and 1455 cm^−1^, 1407 cm^−1^ (region I), which have already been observed in the pristine melamine sample (grey line in Figure 10), are related to the stretching vibrational modes of C=N and C-N moieties, respectively, within the heterocycles [56,61,64]. By considering the main features of the melon computed spectrum (blue line in region I), the contribution due to the -NH and -NH_2_ terminal groups cannot be excluded. Indeed, the feature at 1413 cm^−1^ can be assigned to the bending mode of the C-NH-C units.

From the comparison of the experimental FTIR spectra of pristine melamine and our sample (grey and black experimental lines, respectively, in Figure 10), it becomes apparent that the two main absorptions at 1317 cm^−1^ and 1240 cm^−1^ (region II), which have quite similar intensity, are absent in the precursor, thus confirming that these features are related to partial (blue line) and/or total (red lines) condensed units. Currently, on the basis of the literature data, the 1317 cm^−1^ and 1240 cm^−1^ bands are assigned mainly to the stretching mode of both trigonal C-NH-C units, i.e., the partial-condensation model, or bridging C–N(–C)–C units, which is also called the full-condensation model [6,53,55,64,67,68].

In order to make a more precise assignment, we have to consider the partially condensed melon and the fully condensed g-C_3_N_4_ computed spectra, in which two main features at 1297 cm^−1^ and at 1240 cm^−1^ (blue line) and at 1291 cm^−1^ and at 1262 cm^−1^ (red line) can be observed.

**Scheme 2:**
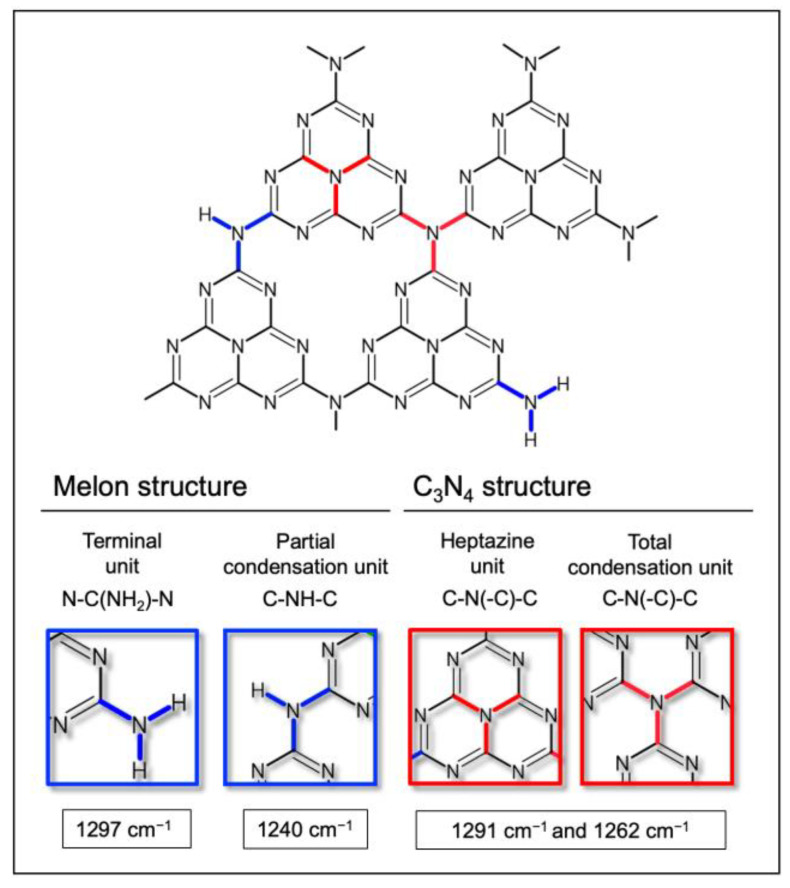
Structural unit models of melon (blue) and C_3_N_4_ (red) with relative computed vibrational frequencies.

According to our model of melon structure, we hypothesise that the feature at 1297 cm^−1^, which shifted downwards with respect to the experimentally observed one at 1317 cm^−1^, can be due to combined collective stretching and bending vibrations of the N-C(NH_2_)-N units, also involving the -NH_2_ terminal groups, while the 1240 cm^−1^ band corresponds mostly to the collective modes of the C-NH-C units involving the –NH groups. Notice that the higher intensity of the 1240 cm^−1^ band with respect to the other components in the 1200–1750 cm^−1^ region of the computed spectrum (blue line) is consistent with our adopted model based on a melon-like structure, which is formed by a sequence of a large number of linked heptazine units. Considering the computed spectrum of the totally condensed system (red line), the features at 1291 cm^−1^ and at 1262 cm^−1^ are both ascribed to the C-N(-C)-C total condensed units either inside the heptazine units or among connected moieties as described in Scheme 2.

Furthermore, the three shoulders observed at about 1195 cm^−1^, 1126 cm^−1^, and 1069 cm^−1^ inside the complex envelope of the experimental spectrum (Region III in Figure 10) can be related to the 1185 cm^−1^, the 1105 cm^−1^, and the 1080 cm^−1^ computed modes of the totally condensed system (red line). From this, it comes out that the shoulders can be assigned to combined vibrations of domains formed by heptazine units and C-N(C)-C condensed moieties, which connect three heptazine units. It is noteworthy that on the melon computed spectrum (blue line), these modes are absent.

Lastly, the sharp peaks at 885 cm^−1^ and 804 cm^−1^ on the experimental spectrum (Region IV) have been assigned to the typical in-plane and out-of-plane bending vibrations (breathing modes) of the heptazine/triazine units [4,6,53,54,55,56,61,62,64,65,66,67]. These features are also found at 880 cm^−1^ and 730 cm^−1^ and at 900 cm^−1^ and 747 cm^−1^ on the computed spectra of the partially condensed melon (blue line) and fully condensed system (red line), respectively.

It is noteworthy that, moving from the melamine precursor spectrum (grey line) to the other spectra (both computed and experimental ones), the different intensity ratio between the (3000–3500 and 1400–1600 cm^−1^) main regions can be justified with a different amount of the terminal units (NH_2_) with respect to the number of heterocycle rings. Namely, this provides a further explanation of the condensation of triazine units in more complex structures to form a heptazine lattice.

In conclusion, from a detailed analysis of FTIR spectra, significant information on the polymerisation degree of carbon nitride-based systems can be obtained. In particular, features due to NH/NH_2_ stretching vibrations, such as wide absorptions in the 1200–1750 cm^−1^ range, which are typical of heterocycle C-N/C=N stretching vibrations, together with the sharp peaks in the 700–800 cm^−1^ range due to breathing modes of the heptazine rings, are indicative of a complex structure consisting of totally condensed g-C_3_N_4_ domains embedded in a partially condensed system, i.e., the melon-string structure formed by a network of linked heptazine units [22,60].

## 5. Conclusions

In summary, polymerised carbon nitride systems have been obtained via a facile one-step calcination method by starting from melamine as an affordable precursor.

The structure analysis based on X-ray powder diffraction makes it possible to highlight the typical layered structure of the semiconductor, while the computational study makes it possible to identify a mixture of both the partially and totally polymerised structures, as expected from the adopted experimental synthesis conditions. Moreover, the geometry optimisation and the topological analysis of the charge density at the PBE-D3/621pol level confirm the tendency of the material to maximise the interactions between layers and rows for both the models.

Combined computation and experimental UV-Vis studies of the electronic properties agree that the material has an indirect band gap with energy values of about 2.59 eV (experimental one), while the theoretical ones are 2.36 eV for the partially polymerised structure and 1.72 eV for the totally condensed g-C_3_N_4_. The experimental band gap value is likely the result of a complex situation in which more and less condensed motifs, including condensed melamine units, small g-C_3_N_4_ condensed domains, etc., are all embedded in a framework of partially condensed strings, which are formed by a network of linked heptazine units, that is, the melon structure.

In addition, a careful analysis of the FTIR features compared to the computed spectra provides significant information on the frequency values of different functional groups and lattice modes on both systems, thus making it possible to highlight the simultaneous presence of domains with different polymerisation levels inside our synthetised specimen.

In future studies, the concept of a mixed system (based on highly condensed g-C_3_N_4_ domains embedded in a less condensed “melon-like” framework) that was developed in this work will be taken into consideration in order either to explore different synthesis and post-synthesis procedures or to design challenging heterostructures, such as C_3_N_4_/C_3_N_4_ homojunctions and/or C_3_N_4_/semiconductor heterojunctions, which could be effective in several renewable-energy-activated processes.

## Figures and Tables

**Table 1:** Structural parameters of partially condensed melon unit cell. Lattice parameters: a, b, c [Å] unit cell vectors; 𝛼, 𝛽, 𝛾 [°] unit cell angles. Volume of unit cell (V [Å^3^]), inter-layer distance [Å], and bending angles before (Fina et al.) and after PBE-D3/621pol optimisation [°]. The difference in percentage between the two structures is reported in the last column.

	Fina et al.	PBE-D3	Difference [%]
a [Å]	16.24	15.18	−6.53
b [Å]	12.52	12.60	+0.64
c [Å]	6.52	6.55	+0.46
𝛼 = 𝛽 = 𝛾 [°]	90	90	0
V [Å^3^]	1324.90	1253.61	−5.38
Inter-layer distance [Å]	3.28	3.26	−0.61
Bending angle N77–N86–N130 [°]	175.0	150.5	−14.0

**Table 2:** Structural parameters of fully condensed g-C_3_N_4_ unit cell. Lattice parameters: a, b, c [Å] unit cell vectors; 𝛼, 𝛽, 𝛾 [°] unit cell angles. Volume of unit cell (V [Å^3^]), inter-layer distance [Å], and bending angles before (Teter and Hamley [35]) and after PBE-D3/621pol optimisation [°]. The difference in percentage between the two structures is reported in the last column.

	Teter and Hamley	PBE-D3	Difference [%]
a [Å]	7.11	6.99	−1.75
b [Å]	7.11	6.99	−1.72
c [Å]	6.49	6.43	−0.99
𝛼 [°]	90.00	77.58	−13.80
𝛽 [°]	90.00	96.17	+6.86
𝛾 [°]	120.00	120.01	0.00
V [Å^3^]	284.37	265.47	−6.64
Inter-layer distance [Å]	3.25	3.12	−4.00
Bending angle N6 –C21 –N3 [°]	180.00	163.90	−8.94

## Data Availability

The data presented in this study are available on request from the corresponding author.

## Supplementary Materials

The following supporting information can be downloaded at: https://www.mdpi.com/article/10.3390/ma16103644/s1, Table S1: Lattice parameters and energy gap of melon system computed with different functionals: PBE-D3, PBE0-D3 and HSE06-D3; Figure S1: Graphical view of bending angles before (175°, on the left side) and after optimisation at PBE-D3 (150.5°, in the centre) and PBE (153.9°, on the right side) level; Figure S2. Melon structure optimised at PBE level and relative structural parameters; Figure S3. Comparison of XRD patterns: experimental (black), obtained from Fina et al. model before and after optimisation (blue line), obtained from Teter and Hamley model before and after optimisation (red line). Figure S4. Reaction scheme of direct heating of melamine precursor; Figure S5. UV-vis spectra of melamine (a); the computed Tauc’s plots for indirect (b) and direct (c) band gap estimation.
