# Supplementary material for: Combined DFT-D3 Computational and Experimental Studies on g-C3N4: New Insight into Structure, Optical, and Vibrational Properties
Source: Materials (Basel). 2023 May 10;16(10):3644. doi: 10.3390/ma16103644 (PMC10220934; doi:10.3390/ma16103644)
Supplement: Supplementary file 1 [file materials-16-03644-s001.zip › materials-2333868-supplementary.pdf]

Combined DFT-D3 Computational and Experimental Studies on g-C_3N_4: New Insight into Structure, Optical, and Vibrational Properties

Paolo Negro*, Federico Cesano, Silvia Casassa and Domenica Scarano*

Department of Chemistry and NIS (Nanostructured Interfaces and Surfaces) Interdepartmental Centre, University of Torino & INSTM-UdR Torino, Via P. Giuria 7, 10125 Torino, Italy

Corrrespondence: pa.negro@unito.it (P.N.); domenica.scarano@unito.it Tel.: +39-011-6707834 (D.S.)

Table S1. Lattice parameters and energy gap of melon system computed with different functionals: PBE-D3, PBE0-D3 and HSE06-D3.

	PBE-D3	PBE0-D3	HSE06-D3
a [Å]	15.18	14.81	14.83
b [Å]	12.60	12.54	12.55
c [Å]	6.55	6.57	6.61
$\alpha=\beta=\gamma$ [°]	90	90	90
V [Å^3]	1253.61	1221.42	1230.75
Eg [eV]	2.36	4.25	3.61

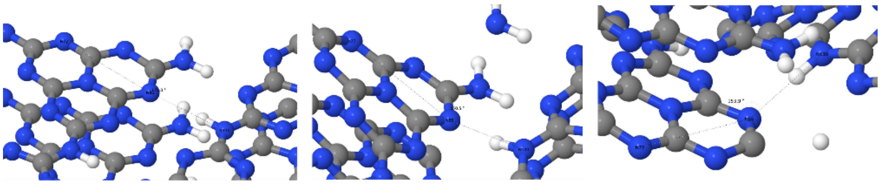

Figure S1. Graphical view of bending angles before (175°, on the left side) and after optimization at PBE-D3 (150.5°, in the center) and PBE (153.9°, on the right side) level.

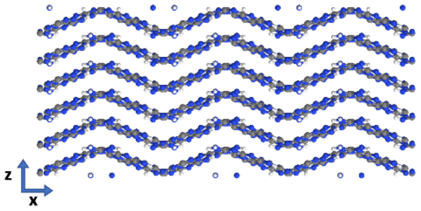

	PBE	Difference Exp-Teo [%]
a [Å]	15.48	4.68
b [Å]	12.64	-0.96
c [Å]	6.89	-5.67
$\alpha=\beta=\gamma$ [°]	90	0
V [Å^3]	1349.05	-1.82
Inter-layer distance [Å]	3.45	-5.18
Bending angle [°] $N_{77}-N_{86}-N_{130}$	153.9	12.06

Figure S2. Melon structure optimized at PBE level and relative structural parameters.

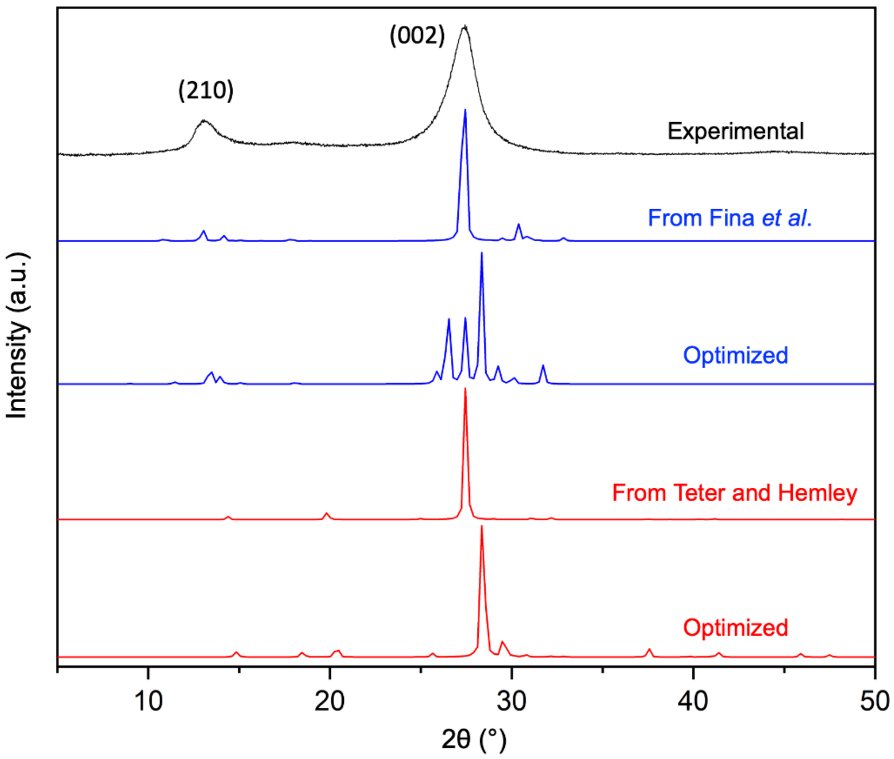

Figure S3. Comparison of XRD patterns: experimental (black), obtained from Fina et al. model before and after optimization (blue line), obtained from Teter and Hamley model before and after optimization (red line).

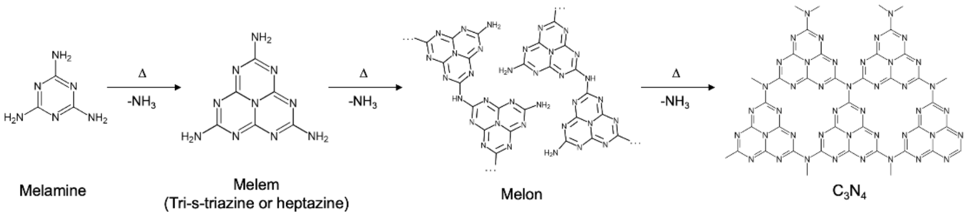

Figure S4. Reaction scheme of direct heating of melamine precursor.

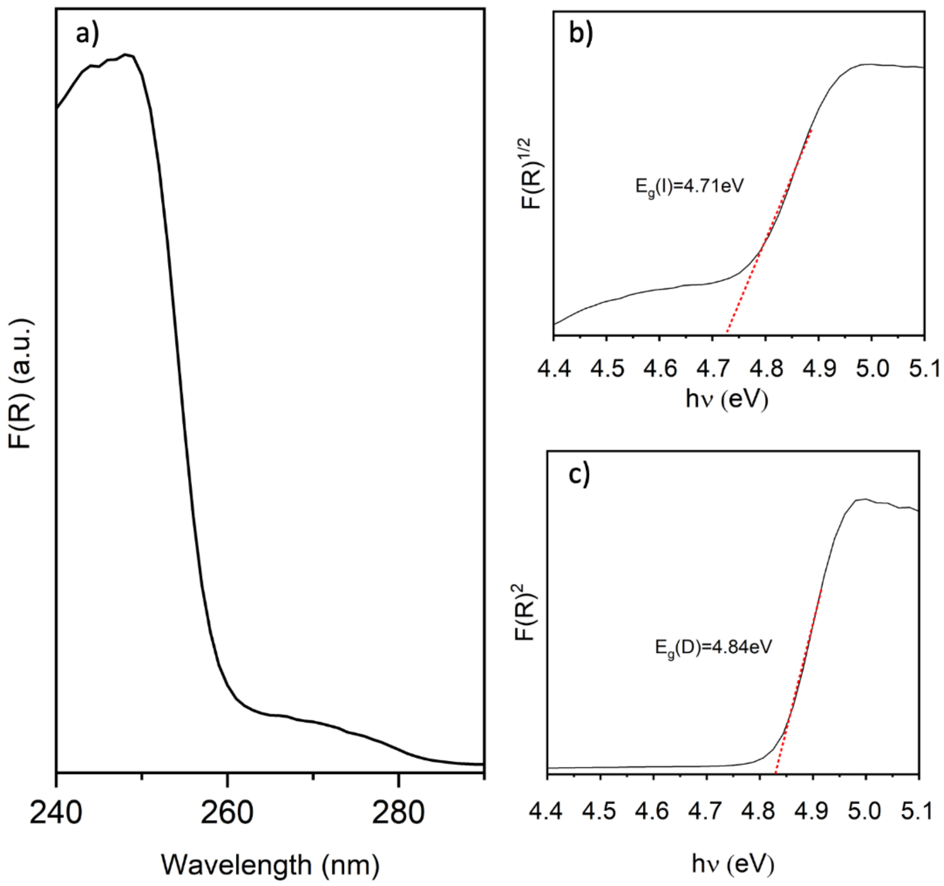

Figure S5. UV-vis spectra of melamine (a); the computed Tauc's plots for indirect (b) and direct (c) band gap estimation.
